# Understanding the characteristics of mass spectrometry data through the use of simulation

**Published:** 2007-02-18

**Authors:** Kevin R. Coombes, John M. Koomen, Keith A. Baggerly, Jeffrey S. Morris, Ryuji Kobayashi

**Affiliations:** 1 Departments of Biostatistics and Applied Mathematics and; 2 Molecular Pathology, University of Texas M.D. Anderson Cancer Center, Houston TX 77030 USA

**Keywords:** mass spectrometry, MALDI, SELDI, simulation, peak capacity, peak quantification, mass resolution, isotope distribution

## Abstract

**Background:**

Mass spectrometry is actively being used to discover disease-related proteomic patterns in complex mixtures of proteins derived from tissue samples or from easily obtained biological fluids. The potential importance of these clinical applications has made the development of better methods for processing and analyzing the data an active area of research. It is, however, difficult to determine which methods are better without knowing the true biochemical composition of the samples used in the experiments.

**Methods:**

We developed a mathematical model based on the physics of a simple MALDI-TOF mass spectrometer with time-lag focusing. Using this model, we implemented a statistical simulation of mass spectra. We used the simulation to explore some of the basicoperating characteristics of MALDI or SELDI instruments.

**Results:**

The simulation reproduced several characteristics of actual instruments. We found that the relative mass error is affected by the time discretization of the detector (about 0.01%) and the spread of initial velocities (about 0.1%). The accuracy of calibration based on external standards decays rapidly outside the range spanned by the calibrants. Natural isotope distributions play a major role inbroadening peaks associated with individual proteins. The area of a peak is a more accurate measure of its size than the height.

**Conclusions:**

The model described here is capable of simulating realistic mass spectra. The simulation should become a useful tool forgenerating spectra where the true inputs are known, allowing researchers to evaluate the performance of new methods for processing and analyzing mass spectra.

**Availability:**

http://bioinformatics.mdanderson.org/cromwell.html

## Introduction

Mass spectrometry is actively being used to discover disease-related proteomic patterns in complex mixtures of proteins derived from tissue samples or from easily obtained biological fluids such as serum, urine, or nipple aspirate fluid (Paweletz et al., 2000; Paweletz et al., 2001; Wellmann et al., 2002; Petricoin et al., 2002; Adam et al., 2002; Adam et al., 2003; Zhukov et al., 2003; Schaub et al., 2004). These proteomic patterns can potentially be used for early diagnosis, to predict prognosis, to monitor disease progression or response to treatment, or even to identify which patients are most likely to benefit from particular treatments.

A typical data set arising in a clinical application of mass spectrometry contains tens or hundreds of spectra; each spectrum contains tens of thousands of intensity measurements representing an unknown number of protein peaks. Any attempt to make sense of this volume of data requires extensive low-level processing in order to identify the locations of peaks and to quantify their sizes accurately. Inadequate or incorrect preprocessing methods, however, can result in data sets that exhibit substantial biases and make it difficult to reach meaningful biological conclusions (Baggerly et al., 2003; Sorace and Zhan, 2003; Baggerly et al., 2004). The low-level processing of mass spectra involves a number of complicated steps that interact in complex ways. Typical processing steps are as follows.

1. **Calibration** maps the observed time of flight to the inferred mass-to-charge ratio.
2. **Filtering** removes random noise, typically electronic or chemical in origin.
3. **Baseline subtraction** removes systematic artifacts, usually attributed to clusters of ionized matrix molecules hitting the detector during early portions of the experiment, or to detector overload.
4. **Normalization** corrects for systematic differences in the total amount of protein desorbed and ionized from the sample plate.
5. **Peak detection and quantification** is the primary goal of low-level processing; it typically involves an assessment of the signal-to-noise ratio and may involve heights or areas.
6. **Peak matching** across samples is required because neither calibration nor peak detection is perfect. Thus, the analyst must decide which peaks in different samples correspond to the same biological molecule.

The potential importance of the clinical applications of mass spectrometry has drawn the attention of increasing numbers of analysts. As a result, the development of better methods for processing and analyzing the data has become an active area of research (Baggerly et al., 2003; Coombes et al., 2003; Coombes et al., 2004; Hawkins et al., 2003; Lee et al., 2003; Liggett et al., 2003; Rai et al., 2002; Wagner et al., 2003; Yasui, Pepe, et al., 2003; Yasui, McLerran et al., 2003; Zhu et al., 2003). It is, however, difficult to determine which methods are better without knowing the true biochemical composition of the samples used in the experiments. To deal with this problem, we have developed a simulation engine in S-Plus (Insightful Corp., Seattle, WA) that allows us to simulate mass spectra from instruments with different properties. In this article, we first derive the mathematical model of a physical mass spectrometry instrument that underlies our simulation. Next, we use the model to explore some of the low-level characteristics of mass spectrometry data, including the limits on mass resolution and mass calibration, the role of isotope distributions, and the implications for methods of normalization and quantification.

### 1: A physical model of a MALDI-TOF instrument

The mass spectrometry instruments most commonly applied to clinical and biological problems use a matrix-assisted laser desorption and ionization (MALDI) ion source and a time-of-flight (TOF) detection system. Briefly, to run an experiment on a MALDI-TOF instrument, the biological sample is first mixed with an energy absorbing matrix (EAM) such as sinapinic acid or ά-cyano-4-hydroxycinnamic acid. This mixture is crystallized onto a metal plate. (The commonly used method of surface enhanced laser desorption and ionization (SELDI) is a variant of MALDI that incorporates additional chemistry on the surface of the metal plate to bind specific classes of proteins (Merchant and Weinberger, 2000; Tang et al., 2004). The plate is inserted into a vacuum chamber, and the matrix crystals are struck with light pulses from a nitrogen laser. The matrix molecules absorb energy from the laser, transfer it to the proteins causing them to desorb and ionize, and produce a plume of ions in the gas phase. Next, an electric field is applied, which accelerates the ions into a flight tube where they drift until they strike a detector that records the time of flight. A quadratic transformation is used to compute the mass-to-charge ratio (*m*/*z*) of the protein from the observed flight time. The spectral data that results from this experiment consists of the sequentially recorded numbers of ions arriving at the detector (the intensity) coupled with the corresponding *m*/*z* values. Peaks in the intensity plot represent the proteins or polypeptide fragments that are present in the sample.

**Figure 1:**
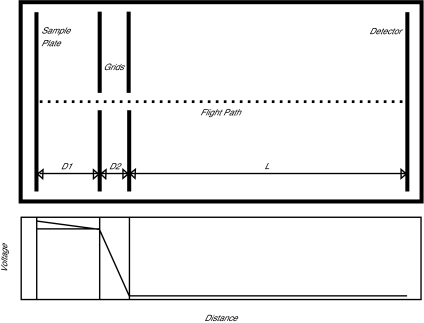
(Top) Simplified schematic of a MALDI-TOF instrument with time-lag focusing. Samples are inserted on a metal plate into a vacuum chamber where they are ionized by a laser. Electric fields between the sample plate and two charged grids accelerate the ions into a drift tube, where they continue until they strike a detector. (Bottom) Voltage potentials along the instrument. The sample plate and grid start at the same potential, but the potential is raised after a brief delay.

We developed code to simulate experiments based on a physical model of a linear MALDI-TOF instrument with time-lag focusing or delayed extraction (Wiley and McLaren, 1955; Vestal et al., 1995). Such an instrument is illustrated schematically in Figure 1. The flight path of a particle in this instrument passes through three regions:

1. **focusing**, from the sample plate to the first grid,
2. **acceleration**, through the electric field between the two charged grids, and
3. **drift**, through the field-free tube from the second grid to the detector.

Our model requires three parameters to describe the lengths of these three regions. We let L denote the length of the drift tube, which is typically on the order of 1 or 2 meters. We let *D*_1_ be the distance from the sample plate to the grid and *D*_2_ the distance between the two grids. These are typically measured in millimeters; the default values used in our simulation are *D*_1_ = 17 mm and *D*_2_ = 8 mm.

At the start of an experiment, the voltage on the sample plate is the same as the voltage on the first grid. By contrast, there is a large potential difference (on the order of *V* = 20000 volts) between the two grids. A laser is fired at the sample, and the matrix molecules absorb energy and transfer it to the sample molecules. The laser imparts different initial velocities to different particles, which we model as a normal distribution, *v*_0_ ~ *N*(*μ*, *σ*), following Beavis and Chait (1991). During this phase of the experiment, the sample ions drift in a field-free zone during a delay time of length *δ*, typically on the order of 600 nanoseconds. After waiting the specified amount of time, the voltage on the sample plate is increased by an amount *V*_1_, typically on the order of 10% of the voltage potential between the charged grids. Different combinations of laser power, delay time, and voltage allow the user to focus the optimum resolution of the instrument for different mass ranges. The electric field resulting from the voltage change causes the positively charged ions to accelerate into the region between the charged grids, where the larger potential difference imparts a larger acceleration. The particles then pass into the drift tube, where they continue to fly until they hit the detector. In our model, the detector counts particles continuously (and perfectly), but it reports the counts at discrete time intervals, with an acquisition time resolution *τ* on the order of a few nanoseconds.

From the description so far, we see that a model of a linear MALDI-TOF instrument with time-lag focusing depends on nine parameters: *L*, *D*_1_, *D*_2_, *V*, *V*_1_, *δ*, *τ*, *μ*, and σ. In a real instrument, the three distance parameters are unchanging characteristics of the design. The user has direct control over the voltages, the delay time, and the acquisition time resolution. The parameters that determine the normal distribution of initial velocities are controlled indirectly by the choice of EAM and by the laser intensity. Since we do not have a good theoretical understanding of how these factors interact to determine the initial velocity distribution, our simulation skips directly to the distribution. By default, we take *μ* = 350 m/sec and *σ* = 50 m/sec, which are compatible with published experimental results (Beavis and Chait, 1991; Juhasz et al., 1997).

We can compute the total time for a particle to travel from the sample plate to the detector as a sum of four contributions: the delay time *δ*, the focus time *t_f_*, the acceleration time *t_a_*, and the drift time *t_d_*, which are calculated below. We assume that all particles start the experiment attached to the sample plate (at *x* = 0) and that the clock starts when the laser is fired. Each particle acquires an initial velocity *v*_0_ ~ *N*(*μ*, *σ*), which is assumed to be independent of the mass. After a delay of length *δ*, the particles are located at position *x*_0_ = *δv*_0_, still traveling at velocity *v*_0_. Using the default value of *δ* = 600 ns and an estimated upper bound on the velocity of 500 m/sec, the particles should be roughly 0.3 mm away from the plate at the end of the delay period

We let *v*_1_ denote the velocity of a particle at the end of the focus phase, when the particle reaches the first grid, and we let *v*_2_ denote the velocity at the end of the acceleration phase, when the particle reaches the second grid and enters the drift tube. It is easiest to understand the final portion of the experiment, during which the particle travels at constant velocity *v*_2_ through a tube of length *L*. So, we have

(1.1) $$L=v_{2}t_{d}$$

During the main acceleration phase, an electric field of voltage *V* accelerates a particle of mass *m* with charge *z* through a distance *D*_2_ by applying a constant force *F*. Because the work W done by the electric field is equal to the change in kinetic energy, we have

(1.2) $$W=zV=FD_{2}=\frac{mv_{2}^{2}}{2}-\frac{mv_{1}^{2}}{2}.$$

Solving equation 1.1 for the velocity and substituting, we find that

(1.3) $$zV=\frac{m}{2}\left(\frac{L^{2}}{t_{d}^{2}}-v_{1}^{2}\right).$$

So,

(1.4) $$t_{d}^{2}=\frac{L^{2}}{2zV/m+v_{1}^{2}}.$$

During the simulation, everything in this equation is known except for the drift time *t_d_* and the velocity *v*_1_ that marks the transition from the focusing phase of the experiment to the acceleration phase.

During the focusing phase, the electric field generated by the potential difference *V*_1_ applies a constant force *F* to the particle. We can determine the force from the work that would be done moving a particle from the sample plate to the first grid, which yields *FD*_1_ = *zV*_1_. During this phase, however, the particle is acclerated through a distance *D*_1_ − *x_0_*, resulting in change of velocity from *v*_0_ to *v*_1_. Using the equality between work and the change in kinetic energy, we find

(1.5) $$\frac{mv_{1}^{2}}{2}-\frac{mv_{0}^{2}}{2}=F(D_{1}-x_{0})=\frac{zV_{1}}{D_{1}}(D_{1}-x_{0}).$$

Solving for the velocity *v*_1_, we find

(1.6) $$v_{1}^{2}=v_{0}^{2}+\frac{2zV_{1}}{mD_{1}}(D_{1}-x_{0}).$$

Since all the quantities on the right hand side of this equation are assumed to be known, we can combine it with equation (1.4) to compute the drift time as

(1.7) $$t_{d}^{2}=L^{2}/\left(\frac{2zV}{m}+\frac{2zV_{1}}{m}\frac{D_{1}-x_{0}}{D_{1}}+v_{0}^{2}\right).$$

We now turn our attention to the time spent in the acceleration and focusing phases of the experiment. During both phases, the particle is subject to a constant force, and so undergoes constant acceleration. In these circumstances, one knows that the change in velocity is equal to the acceleration times the duration. As we have seen, the force during the main acceleration phase is *F* = *zV*/*D*_2_. Combining this with Newton’s Second Law, we have *a* = *zV*/*mD*_2_, so

(1.8) $$t_{a}=\frac{v_{2}-v_{1}}{a}=\frac{mD_{2}}{zV}(v_{2}-v_{1})=\frac{mD_{2}}{zV}\left(\frac{L}{t_{d}}-v_{1}\right).$$

Since *t_d_* can be computed from known values using equation (1.7) and *v*_1_ can be computed using equation (1.6), this allows us to compute the time spent during the acceleration phase.

As we have also seen, the force during the focusing phase is *F* = *zV*_1_/*D*_1_, so the acceleration is *a* = *zV*_1_/*mD*_1_. Thus,

(1.9) $$t_{f}=\frac{v_{1}-v_{0}}{a}=\frac{mD_{1}}{zV_{1}}(v_{1}-v_{0}).$$

In summary, to simulate the flight time of a particle of mass *m* and charge *z*, given the nine parameters describing the setup of the instrument during the experiment, we first sample the initial velocity *v*_0_ from the appropriate distribution. We then compute the position *x*_0_ = *δv*_0_ at the end of the delay phase. Next, we use (1.6) to compute *v*_1_, (1.7) to compute the drift time, (1.8) to compute the acceleration time, (1.9) to compute the focus time, and report the total time of flight as

(1.10) $$TOF=\delta +t_{f}+t_{a}+t_{d}.$$

## Results

We now apply the model described in the previous section, and its S-Plus implementation, to understand some of the fundamental characteristics of mass spectra. In particular, we look at some physical factors that affect the mass resolution (Ingendoh et al., 1994; Barbacci et al., 1997, Vestal and Juhasz, 1998), at limits on the accuracy of mass calibration (Christian et al., 2000; Hack and Benner, 2002), at the role of isotope distributions (Zhang et al., 1997), and at implications for the normalization and quantification of MALDI-TOF data.

### 2: Mass Resolution

Our model contains two factors that affect the mass resolution of the instrument: the acquisition time resolution (or period) of the detector and the distribution of initial velocities. We begin by considering the effect on mass resolution caused by the discretization of time by the detector. As we will see, this effect is, in general, far smaller than that due to the spread in initial velocities. If there were no variability in the initial velocities, then all ions with the same mass and charge would strike the detector at the same instant. In this idealized setting, our ability to distinguish ions of different mass would be completely determined by the period of the detector. We can get a rough estimate of the magnitude of this effect as follows. First, assume that *v*_0_ = 0 and that the dominant component of the time is spent in the drift tube. Then (1.7) simplifies to

(2.1) $$t_{d}^{2}=L^{2}/\left(\frac{2zV}{m}+\frac{2zV_{1}}{m}\right)=\frac{mL^{2}}{2z(V+V_{1})}.$$

Solving for the mass-to-charge ratio *M* = *m*/*z*, we obtain

(2.2) $$M=\frac{m}{z}=t_{d}^{2}\frac{2(V+V_{1})}{L^{2}}.$$

Differentiating with respect to time, we find that

(2.3) $$\Delta M\approx 4t_{d}\Delta t_{d}\frac{V+V_{1}}{L^{2}}.$$

In other words, the absolute mass error arising from using a discrete-time detector grows linearly with the time and is thus proportional to the square root of *M*. Alternatively, we can compute the relative mass error, which satisfies

(2.4) $$\frac{\Delta M}{M}\approx 2\frac{\Delta t_{d}}{t_{d}}.$$

So, the relative mass error is inversely proportional to the time (or to the square root of *M*).

**Figure 2:**
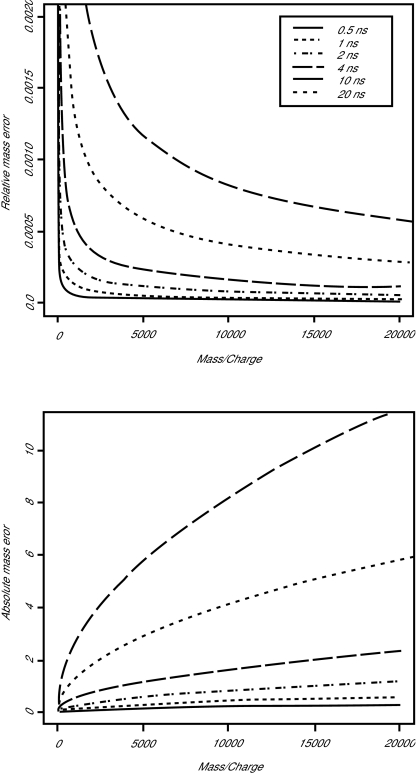
Plots of the relative (top) and absolute (bottom) mass resolution arising from a discrete time detector at four different acquisition periods from 0.5 ns to 20 ns.

In order to interpret equations (2.3) and (2.4) numerically, we measure the mass in Daltons (where 1 Dalton = 1.6603×10^−27^ kg) and the charge in integer multiples of 1.602×10^−19^ coulombs (which is the charge on a single electron or proton). Figure 2 displays, for six different detection periods, the mass resolution in an ideal noise-free instrument. The figure was produced using typical values for the instrument parameters (*L* = 1 m, *D*_1_ = 17 mm, *D*_2_ = 8 mm, *V* = 20000 volts, *V*_1_ = 2000 volts). Shorter detection periods, of course, yield better mass resolution. One should also note that doubling the length of the drift tube is almost equivalent to cutting the detector period in half. At a period of τ = 4×10^−9^ seconds, which is commonly used on a Ciphergen SELDI instrument, the absolute mass error at 20,000 Daltons is less than 2.5 Daltons, which represents a relative error near 0.01%.

**Figure 3:**
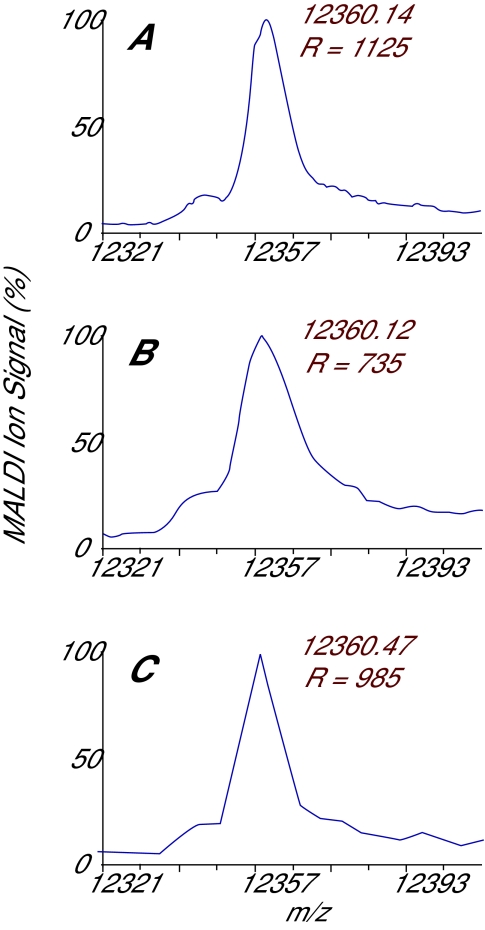
Effect of acquistion period on cytochrome C [M+H]+ with data digitized at (A) 4 ns, (B) 10 ns, and (C) 20 ns. The resolution is close to the theoretical value for 4 ns sampling, decrease significantly at 10 ns sampling, and is artificially enhanced at 20 ns sampling.

We tested these theoretical resolutions by collecting MALDI spectra on a sample containing cytochrome C at three different acquisition periods (Figure 3). The resolution (the reciprocal of the relative mass error) when the acquisition period was set to 4 ns is close to the theoretical value. As expected, the resolution was significantly decreased when acquiring data every 10 ns. Interestingly, the peak appears artificially enhanced when sampling at the slower rate of 20 ns; the apparent sharpness is a direct result of the fact that only three data points are acquired over the main part of the peak.

The relative mass error of actual MALDI-TOF instruments is typically reported in the range of 0.1%, which suggests that factors other than the period of the detector play a larger role. In our model, the most important factor affecting the mass resolution is the distribution of initial velocities; this is the only stochastic factor included in the model. Figure 4 shows the simulated spectra from ions of 3000 and 3003 Daltons, with a mean initial velocity of 350 meters/second as the standard deviation increases from 5 to 30. The twin peaks are easily resolved when the standard deviation is small, but they gradually coalesce into a single broad peak as the standard deviation increases.

## Calibration

Calibration of a MALDI-TOF instrument is performed in order to accurately map the observed time-of-flight to a mass-to-charge ratio. Calibration involves both experimental observations and theoretical computations. Most MALDI-TOF spectra are calibrated externally by running a separate experiment, under the same conditions, using a sample that only contains a small number (typically 5 to 7) of proteins of known mass. Computationally, we simplify the equations of Section 1 by concentrating on the portion of the flight time spent in the drift tube. In this way, we see that *m*/*z* is approximated by a quadratic function of the observed flight time. The unknown coefficients of this quadratic are estimated from the calibration spectrum using least squares.

Even under ideal conditions, the errors in this approximation can become fairly large when the calibration equation is extrapolated beyond the range of masses of the calibrants. We simulated calibration spectra using the default parameters from the previous section for two different calibrant mixes. The first mix contained proteins with masses of 4, 7, 10, 12, and 15 kDa; the second mix contained calibrants with masses of 2, 7, 12, 20, and 35 kDa. We then simulated spectra containing masses from 1 to 50 kDa at 1000 Dalton intervals and determined the observed “calibrated” masses from each of the two mixes. In both cases, the mass of the 1000 Dalton protein was miscalibrated by more than 2%. Calibration errors within the region spanned by the calibrants was typically near 0.1%, but the error started to grow considerably outside the calibrant range (Figure 4). Calibration is more difficult in the presence of a spread of initial velocities, because the location of the peak adds an additional element of error.

To test these results, we ran a MALDI experiment using calibrants at masses close to those used in the two theoretical mixtures (2466 Da, 3660 Da, 7527 Da, 13683 Da, 15054 Da, and 29023 Da). We included other proteins whose masses extended beyond the calibrant range. Specifically, an equimolar protein mixture containing ribonuclease A, serum albumin, carbonic anhydrase II, hemoglobin, ovalbumin, and cytochrome c (all purchased from Sigma, St. Louis, MO) was combined at a 20:1 ratio with the 4700 calibration peptide mixture (Applied Biosystems, Framingham, MA). Aliquots of this solution were mixed 1:1 with sinapinic acid (20 mg/ml) in 50% acetonitrile and 50% aqueous 0.1% TFA. Positive ion MALDI mass spectra consisting of 250 laser shots were acquired in linear mode on an Applied Biosystems Voyager DE-STR. Both myoglobin (m/z 16952) and serum albumin (m/z 66431) were used as standards to optimize the resolution for small proteins and large proteins, respectively. Typical instrument settings for the myoglobin method were 25 kV accelerating voltage, 93% grid voltage, and 700 ns delay; for serum albumin, 25 kV, 91%, and 900 ns were used. Resolution values were calculated by dividing the centroid *m*/*z* value calculated for the peak by the full-width at half maximum (FWHM), using the Data Explorer software which came with the instrument. Four point calibrations were performed using Data Explorer as well, using different peaks. The results, shown in Table 1, indicate that TOF instruments have mass dependent focusing (i.e., optimizing the resolution for a specific *m*/*z* value reduces the resolving power for other *m*/*z* values). The values in the table are typical for our MALDI instrument; values for SELDI are usually lower, by a factor of 2–5. Higher resolution indicates higher data quality, because peak capacity is increased and mass measurement accuracy improves.

### Isotope distributions

We have seen that sharply defined peaks erode quickly into broad hills as the standard deviation of the initial velocity distribution increases. Even with fairly small values for the standard deviation, it can be difficult to resolve peaks whose mass differs by a single Dalton. In practice, however, even a pure solution of a single protein includes molecules whose mass differs by one Dalton. The reason, of course, is the existence of naturally occurring stable isotopes of common elements [Zhang et al., 1997]. Only 98.89% of naturally occurring carbon atoms are in the form of ^12^C; most of the remaining 1.11% consists of atoms of ^13^C. In the same way, ^14^N accounts for 99.63% of naturally occurring nitrogen, with the remaining atoms in the form of ^15^N. Oxygen exists in three stable isotopes, with ^16^O accounting for 99.76% of atoms, ^18^O for 0.20%, and ^17^O for 0.04%. These three elements account for most of the isotope differences between protein molecules (with the possible addition of a few sulfur molecules).

Our simulation includes the isotope distributions of individual proteins. By assuming that most of the mass of a protein is accounted for by the atoms of carbon, nitrogen, and oxygen (with their numbers in proportions of about 6:2.5:1), we can get a crude approximation of the number of atoms that might occur as heavier isotopes by dividing the nominal mass by 15. We then model the process of incorporating heavier isotopes using a binomial distribution with a success rate of 0.0111. We make another simplification by assuming that a heavier isotope always adds one to the mass (which downweights the less abundant oxygen atoms).

**Figure 5:**
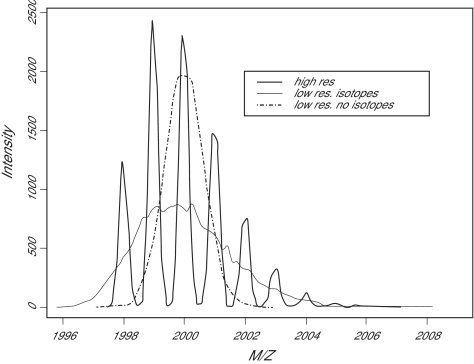
The effect of the isotope distribution on the size and shape o peaks. Peaks on a low resolution instrument are expected to be lower and broader after accounting for isotopes.

Figure 5 illustrates how accounting for the isotope distribution of a peak at 2000 Daltons lowers and broadens the peak shape. This effect becomes more pronounced at higher masses because there are more chances for a larger molecule to incorporate different isotopes. We can estimate the magnitude of the effect using the same simplifications we have incorporated in our model. The distribution of the number of heavier isotopes in a protein of mass *m* is approximated by a binomial distribution, Binom(*m*/15, 0.0111), and so the expected number of heavier isotopes is 0.0111*m*/15 = 0.00074*m*. There is still notable skew in the distribution in the mid-mass range. When *m* is large, however, the distribution is approximately normal with standard deviation √(*m*/15)(0.0111) (0.9889) = 0.027 √*m*. To illustrate this result, when m = 20000 Daltons, we expect to see an average of about 15 heavier isotopes per molecule, with 99% of molecules containing between 3 and 27 heavier isotopes. This effect spreads the peak over a range of at least 24 Daltons or about 0.012% of the nominal mass. The offset of the center of the peak can also affect the calibration and the interpretation of the results.

### Quantification

**Figure 6:**
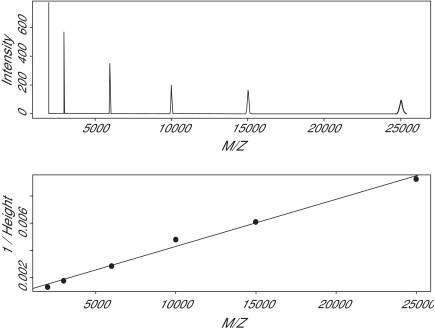
(Top) A simulated spectrum containing equal numbers of molecules of six different proteins, with masses equal to 2, 3, 6, 10, 15, and 25 kDa. (Bottom) The reciprocal of the height of the peak is approximately a linear function of the mass.

As we pointed out in the introduction, the primary goal of the low-level analysis of a single mass spectrum is to locate and quantify the peaks that correspond to individual proteins. We will not discuss peak finding in this paper since we have addressed this issue elsewhere (Coombes et al., 2004), but we will consider the problem of quantifying peaks after they have been found. Two natural candidates for the size of a peak are its height and its area. We simulated a spectrum containing equal numbers of molecules of six different proteins over the mass range from 2000 to 25000 Daltons (Figure 6). In this idealized noise-free setting, the areas of the peaks are, as expected, equal. The heights, however, decrease as the mass increases, which is consistent with what we have already seen about the resolution. Interestingly, the height appears to be inversely proportional to the mass.

The fact that peak areas accurately reflect the number of molecules of a given protein species that hit the detector in an ideal instrument suggests that the common normalization strategy of dividing by the total ion current (the area under the curve) is a reasonable way to account for differences in the total amount of sample protein that was applied to the sample plate.

## Discussion

In this article, we have described some preliminary results using a simulation of mass spectra based on a physical model of a linear MALDI-TOF instrument with time-lag focusing. We have shown that our simulation recovers some of the important characteristics of real data. We expect the simulation to be a useful tool in developing improved methods for processing and analyzing mass spectrometry data, since it will allow us to generate complex spectra where the true locations and sizes of the peaks are known. The simulation code in S-Plus is available from our web site (http://bioinformatics.mdanderson.org/cromwell.html).

Some recent analyses of SELDI spectra have used every measured time point (or every *m*/*z* value) as a potential feature used to build a classifier to distinguish cancer samples from normal samples (Petricoin et al., 2002; Zhu et al, 2003). We believe that this approach is misguided. As we have seen, the spread in initial velocities and the isotope distribution can cause the measurement of a single protein to extend over many time points. From a biophysical perspective, it seems unreasonable to treat things that cannot possibly be distinguished within the resolution of the instrument as independent entities. From a statistical perspective, complications arise when one treats highly correlated measurements as though they were independent. These statistical difficulties are compounded by the tremendous amount of multiple testing that accompanies the selection of a few features out of several thousand *m*/*z* values.

We can perform a simple calculation to get an idea of how many peaks can be resolved in a spectrum. The “peak capacity” of a mass spectrum is defined as the maximum number N of peaks could be distinguished as *m*/*z* ranges between a and b with a relative mass error *r*. Given perfect spacing of the peaks, the first peak would be found at *a*, the second at *a* + *ra* = (1 + *r*)*a*, the third at (1 + *r*)^2^*a* and the last at *b* = (1 + *r*)*^N^*^+1^*a*. Thus, we can compute the peak capacity explicitly as

$$N=-1+\frac{log(b/a)}{log(1+r)}.$$

As an example, when *a* = 2000, *b* = 20000, and *r* = 0.1%, then the peak capacity is *N* = 2303. If the relative mass error degrades to 0.2%, then the peak capacity diminishes to only 1152, and with a relative mass error of 0.5%, the peak capacity is only 461. It is interesting to note that, in our experience with SELDI data in this mass range, we can typically identify between 100 and 200 peaks.

Although we have talked about quantification of peaks in this article, we should make it clear exactly what is being quantified. Our simulation models those molecules that are desorbed from the surface, ionized, and detected. Being able to accurately quantify these molecules is only part of the problem. In an actual experiment, we would like to be able to quantify the number of molecules of each mass that were deposited on the surface. Unfortunately, the selection process that determines which molecules make it off the surface involves extremely complicated chemical interactions between the analytes and the matrix and among the analytes. We can (potentially) succeed in making mass spectrometry quantifications more precise, but are likely to be stymied in efforts to make them more accurate.

There are a number of potential enhancements to the simulation model described here. First, the current model ignores the physics of the detector, which is another source of stochastic noise in the system. Second, one could try to model a more elaborate instrument. The obvious next step would be to include a reflectron instead of a simple linear flight path. Third, we could extend the modeling of the isotope distribution to consider other common molecule alterations, such as the inclusion of salt adducts or the loss of water molecules. Fourth, one might want to explicitly model some of the interactions of the particles in the plume before the focusing electric field is imposed.

Our eventual goal is, as mentioned above, to simulate complex mixtures of proteins in order to evaluate the behavior of different methods to process and analyze mass spectra. These simulations are likely to require additional stochastic components beyond the distribution of initial velocities and of isotopes. For example, it is reasonable to believe that the voltage is essentially constant for all molecules in a single experiment. When simulating experiments with different samples across laboratories or across time, however, variability in the nominal voltages (or in the specified delay time) is an additional source of noise. One will also have to decide how to simulate the background electronic or chemical noise and realistic baseline curves. Finally, there is the challenge of deciding what kinds of truth (number, mass, intensity, and variability of peaks) to simulate for the input to the present simulation engine. We believe that these challenges will eventually be met, and that the simulation tool described in the present article is a useful step in the right direction.

## Figures and Tables

**Figure 4a:**
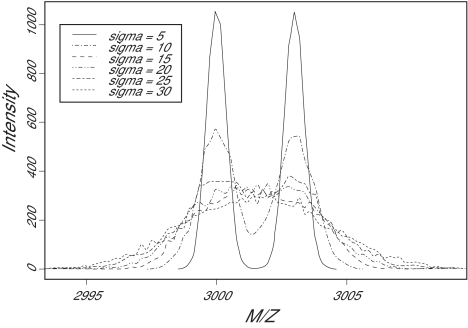
Simulated peaks at 3000 and 3003 Daltons with different values for the standard deviation of the initial velocity. As the standard deviation increases, the resolution decays rapidly

**Figure 4b:**
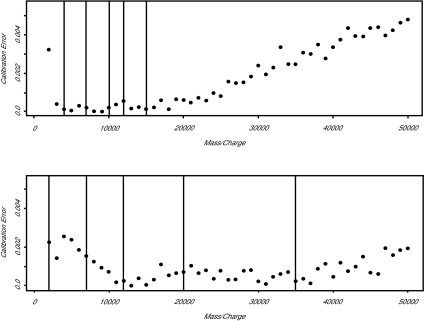
Plot of the relative calibration error (as a percentage of the mass) for two different mixtures of calibrants. Vertical lines in each plot indicate the masses of the five calibrants. Calibration error increases rapidly outside the mass range spanned by the calibrants.

**Table 1:** Resolution as a function of mass, optimizing for two different regions.

		Optimized for Myoglobin		Optimized for albumin	
Ion	M/Z	Singe Spectrum	Average of 4	Single Spectrum	Average of 4
ACTH 18–39	2466.72	300	289	146	156
ACTH 7–38	3660.19	351	248	134	168
RNase A 2+	6842.11	634	680	180	180
HBA 2+	7257.59	675	573	192	216
HBB 2+	7978.70	711	606	143	152
RNase A	13683.23	972	826	239	274
HBA	15054.18	864	748	263	267
HBB	15956.39	626	386	189	207
CAH2	29023.59	222	207	277	331
BSA	66431.00	60	55	69	63
